# Cellulose-Enriched Microbial Communities from Leaf-Cutter Ant (*Atta colombica*) Refuse Dumps Vary in Taxonomic Composition and Degradation Ability

**DOI:** 10.1371/journal.pone.0151840

**Published:** 2016-03-21

**Authors:** Gina R. Lewin, Amanda L. Johnson, Rolando D. Moreira Soto, Kailene Perry, Adam J. Book, Heidi A. Horn, Adrián A. Pinto-Tomás, Cameron R. Currie

**Affiliations:** 1 Department of Energy Great Lakes Bioenergy Research Center, University of Wisconsin-Madison, Madison, Wisconsin, United States of America; 2 Department of Bacteriology, University of Wisconsin-Madison, Madison, Wisconsin, United States of America; 3 Centro de Investigación en Estructuras Microscópicas, Universidad de Costa Rica, San José, Costa Rica; 4 Centro de Investigación en Enfermedades Tropicales, Universidad de Costa Rica, San José, Costa Rica; 5 Departamento de Bioquímica, Facultad de Medicina, Universidad de Costa Rica, San José, Costa Rica; 6 Centro de Investigación en Biología Celular y Molecular, Universidad de Costa Rica, San José, Costa Rica; University Paris South, FRANCE

## Abstract

Deconstruction of the cellulose in plant cell walls is critical for carbon flow through ecosystems and for the production of sustainable cellulosic biofuels. Our understanding of cellulose deconstruction is largely limited to the study of microbes in isolation, but in nature, this process is driven by microbes within complex communities. In Neotropical forests, microbes in leaf-cutter ant refuse dumps are important for carbon turnover. These dumps consist of decaying plant material and a diverse bacterial community, as shown here by electron microscopy. To study the portion of the community capable of cellulose degradation, we performed enrichments on cellulose using material from five *Atta colombica* refuse dumps. The ability of enriched communities to degrade cellulose varied significantly across refuse dumps. 16S rRNA gene amplicon sequencing of enriched samples identified that the community structure correlated with refuse dump and with degradation ability. Overall, samples were dominated by Bacteroidetes, Gammaproteobacteria, and Betaproteobacteria. Half of abundant operational taxonomic units (OTUs) across samples were classified within genera containing known cellulose degraders, including *Acidovorax*, the most abundant OTU detected across samples, which was positively correlated with cellulolytic ability. A representative *Acidovorax* strain was isolated, but did not grow on cellulose alone. Phenotypic and compositional analyses of enrichment cultures, such as those presented here, help link community composition with cellulolytic ability and provide insight into the complexity of community-based cellulose degradation.

## Introduction

The complex polysaccharides stored in the plant cell wall are the most abundant source of organic carbon in terrestrial ecosystems [1]. Select lineages of bacteria and fungi have evolved the ability to enzymatically deconstruct the primary component of plant cell walls, cellulose, a crystal of β-1,4-linked glucose molecules [1,2]. These microbes are critical for driving the terrestrial carbon cycle. Furthermore, they are a valuable resource to identify cellulase enzymes for the sustainable, economical production of cellulosic biofuels [3].

The enzymes used to break down cellulose are well characterized for a small set of microbial isolates [4–9]. However, in natural systems, organisms degrade plant biomass within communities [10–14]. Interactions between species influence cellulose degradation [4,15,16], but the complexity of natural systems hinders a full understanding of how plant biomass break down is altered by microbial interactions and by the underlying diversity of communities. Enrichments of environmental samples with either cellulose or plant biomass as the sole carbon source are an effective method to select for the portion of microbial communities capable of plant biomass degradation [10,17–19]. Critically, this method preserves the community interactions necessary for cellulose break down, allowing for the analysis of cellulolytic organisms within a community and for insight into the ecology of cellulolytic communities as a whole.

**Fig 1:**
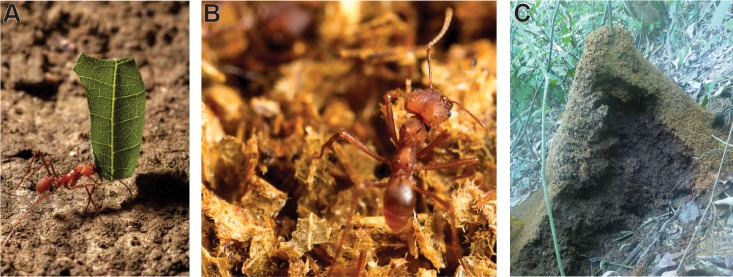
Leaf-cutter ants concentrate decaying plant matter in refuse dumps. **(A)** Leaf-cutter ants are dominant herbivores in Central and South American rain forests. An *Atta* worker carries a leaf fragment back to her nest in Costa Rica. (**B)** The leaf structure is still visible in the dump material tended by this *Atta* worker in the Currie lab at University of Wisconsin-Madison. (**C)** A vertically cross-sectioned *A*. *colombica* refuse dump in Costa Rica. **Photo credits:** Don Parsons (A, B), Gina Lewin (C).

In Central and South American tropical forests and savannahs, leaf-cutter ants and their symbiotic microbes are dominant herbivores and therefore important for carbon cycling. An individual mature leaf-cutter ant colony harvests hundreds of kilograms of leaf material per year (Fig 1A) [20]. In tropical savannahs, these ants are estimated to harvest as much as 13–17% of total leaves produced [21]. Harvested leaves are partially decomposed in subterranean chambers by a mutualistic fungus that the ants cultivate as their food source [22]. However, the fungal cultivar only degrades ~50% of the total leaf material including only 30% of the cellulose in the leaves [23]. Ants move the remaining, cellulose-enriched leaf material to refuse dumps (Fig 1B and 1C). These dumps function as compost piles where complex microbial communities drive the degradation of the cellulose-rich recalcitrant plant material [24,25].

Mature colonies of the leaf-cutter ant species *Atta colombica* maintain a large, aboveground refuse dump downslope from the main nest [20,26]. A single colony of up to 2 million worker ants can dispose of more than 100 refuse particles per minute [27], totaling to over one hundred kilograms (wet weight) of refuse material per year [20]. As material accumulates, the refuse dump can grow to be over one meter in height and two meters in diameter, acquiring vertical stratification with the freshest material in the top strata and the oldest, most recalcitrant material in the bottom strata [26]. Similar to human-produced compost piles, there are high levels of metabolic activity within refuse dumps as microbes degrade the cellulose and other recalcitrant material deposited by the ants [26,28]. The concentration of cellulose quickly decreases from 110 μg/ml in the bottom of the fungus garden to 43 μg/ml in the top strata of the dump to 30 μg/ml in the lower strata of the dump [23,25]. Refuse dumps are also enriched for nitrogen, phosphorus, and other nutrients compared with the nutrient-poor tropical soil [26,28]. However, microorganisms deplete these nutrient levels to those of surrounding soil within one year of a colony dying or moving locations [28].

Culture-independent work has demonstrated that there is a highly diverse but unique community of microbes within refuse dumps dominated by Proteobacteria, Actinobacteria, and Bacteroidetes [24,25]. The microbial community structure of refuse dumps is generally similar among colonies. However, community succession occurs between the upper, middle, and lower strata of the dump piles, reflecting differences in abiotic properties such as oxygen levels (more anaerobic in lower layers) and biomass composition (more recalcitrant in lower layers) [25]. Additionally, the refuse dump community differs from the *Enterobacteriaceae*-dominated community in fungus gardens [24,29] and from the Acidobacteria- and Proteobacteria-dominated community found in the nutrient-depleted tropical forest soil [30,31]. Overall, the refuse dump microbial community is well defined by these analyses, but its complexity has prevented a clear understanding of the organisms that may contribute to cellulose degradation.

Here, we analyzed microbial communities in leaf-cutter ant refuse dumps to study plant biomass degradation within a community context. We used electron microscopy to observe the degradation of plant cells and microbial communities in the leaf-cutter ant refuse dump. Then, to analyze simple communities capable of cellulose degradation, we performed enrichments on cellulosic filter paper and measured the ability of microbial communities to degrade cellulose across three layers of five *A*. *colombica* refuse dumps. We identified the microbial community composition in a range of samples using 16S rRNA gene amplicon sequencing. The alpha diversity of each sample and the beta diversity between samples were calculated to identify patterns in the community structure that correlated with degradation ability, ant colony, or refuse dump layer. Additionally, we isolated the most abundant community member detected across samples, an *Acidovorax* sp., and analyzed its ability to degrade cellulose. Through these methods, we identified community members that are important for cellulose degradation, and we improved our understanding of the interplay between microbial community composition and the ability to degrade cellulose.

## Materials and Methods

### Sample Collection

All samples used for this study are covered by the Resolution Number 009, from the Comisión Institucional de Biodiversidad of the University of Costa Rica, and no protected species were sampled in this study. Samples for electron microscopy were aseptically collected from the top of *A*. *colombica* refuse dumps in May 2010 and in April 2011 with permission on private land in La Palma, Osa, Costa Rica. Dump fragments were immersed in Karnovsky fixative (2.5% glutaraldehyde and 2% paraformaldehyde in 0.1 M phosphate buffer, pH 7.4) and kept at 4°C for transportation to the Center of Research in Microscopic Structures at the University of Costa Rica.

For enrichments, the top, middle, and bottom layers of five *A*. *colombica* colonies were aseptically collected in July 2012 on protected land at Carara National Park, Costa Rica. These collections were permitted by Resolution ACOPAC-INV-006-10 from the Área de Conservación Pacífico Central, Sistema de Áreas de Conservación of the Ministerio del Ambiente, Energía y Telecomunicaciones. *A*. *colombica* dumps were cross-sectioned vertically, with the material on the exterior of the dump collected as “top”, material in the middle third designated as “middle”, and material in the bottom third labelled as “bottom” (S1A Fig). Dumps were all within 500 m of each other. All material was stored at 4°C.

### Electron Microscopy

To analyze the ultrastructure of *A*. *colombica* refuse dumps and look for the presence of microorganisms, we used scanning and transmission electron microscopy (SEM and TEM, respectively) techniques (n = 2 for SEM and n = 2 for TEM). Samples in Karnovsky fixative were left at 4°C overnight. Samples were post-fixed in 1% osmium tetroxide for at least 1 hour and dehydrated with ethanol (SEM; 30%, 50%, 70%, 80%, 90%, 95%, 100%) or acetone (TEM; 30%, 50%, 70%, 90%, 100%). SEM samples were dried by sublimation in a freeze dryer (VFD-20, VD Inc.) after immersion in tert-butanol. Dry samples were mounted on aluminum stubs, coated in gold with an Ion coater (IB-3, Giko), and examined on Hitachi S-570, S-2360N, and S-3700N electron microscopes. TEM samples were infiltrated with epoxy resin (Spurr) after dehydration and sectioned with a PT-PC PowerTome ultramicrotome (RMC products). Sections (70 nm) were stained with uranyl acetate (4% in 50% ethanol) and 2% Sato’s Triple lead and examined on Hitachi H-7000 and H-7100 transmission electron microscopes.

### Overview of Enrichment Design

From each layer of each refuse dump, six pieces of approximately 3 mg (~2 mm diameter) of refuse dump material were inoculated into individual test tubes containing 5 mL of media and a 1x10 cm strip of Whatman #1 filter paper pressed against the side of the tube as the sole carbon source (S1B Fig). M63 minimal medium was used, containing in 1 L: 61.5 mM potassium phosphate dibasic (Acros, Geel, Belgium), 38.5 mM potassium phosphate monobasic (Acros, Geel, Belgium), 15.1 mM ammonium sulfate (Gibco, Grand Island, NY), 0.5 mL of an iron solution (1 mg/ml iron sulfate in 0.01 M HCl), 1 mL of 1M magnesium sulfate solution, 1 mL of 1 mg/ml thiamine solution (Acros, Geel, Belgium), and 5 mL of SPV-4 trace elements solution [32]. These filter paper test tubes are a useful tool for determining the ability of a microbial community to grow on cellulose; aerobic cellulolytic communities grow directly on the filter paper and eventually break it into two pieces at the air/liquid interface. Additionally, the minimal media conditions were chosen to limit nutrients available to the microbes, requiring the degradation of cellulose for carbon and encouraging cross-feeding between community members. Samples were grown at 30°C (to replicate the temperature of the refuse dumps when samples were collected), shaking at 250 rpm. After 14 days, samples were vortexed, and 200 μL was transferred into two sets of tubes: (1) three qualitative tubes each containing a strip of filter paper to determine how many days were necessary to break the filter paper and (2) three quantitative tubes with pre-weighed, submerged filter paper to compare the percentage of cellulose degraded across samples (S1C Fig). As detailed below, this combination of qualitative and quantitative cultures allowed us to analyze the community composition and the cellulolytic ability of the enrichment communities, while ensuring that the high concentrations of nutrients from the original dump material did not influence our data.

Qualitative tubes contained a strip of 1x10 cm filter paper in M63 minimal media. Cultures were grown at 30°C, 250 rpm and were checked daily for visible signs of growth on the filter paper or break down of the filter paper. Three days after the filter paper broke in half, samples were vortexed, and 1.5 mL samples were collected for future DNA extraction and amplicon sequencing (S1D Fig). This three day time point was chosen to provide a representative sample of the cellulose-degrading community, while allowing for enough biomass for DNA extraction and sequencing. For communities where the filter paper did not break, samples for DNA extraction were collected after 14 days. DNA samples were centrifuged in a benchtop centrifuge at 16,100 x g for 10 min, the supernatant was removed, and the cell pellets were frozen at -20°C.

Quantitative tubes contained two 1x4 cm strips of pre-weighed filter paper and 8 mL of M63 media. Controls contained filter paper with no inoculum. Cultures were grown shaking at 250 rpm at 30°C for 10 days. A single time point was used for quantitative tube sampling to allow for comparison across all samples; ten days provided of a wide range of degradation values. The percentage of cellulose degraded was measured using a previously published acid detergent-based method [33,34].

Samples were named based on the refuse dump (colony 1–5), followed by the layer, followed by the inoculation replicate (A-F). For example, inoculation A from the top layer of dump 2 is “2 Top A”. No data were collected for samples 4 Middle A and 4 Bottom C because of technical difficulties.

### DNA Extraction

DNA was extracted using the PowerSoil® DNA Isolation Kit (MoBio, Carlsbad, CA) with the following modifications. PowerBeads were added to the microcentrifuge tube with the thawed cell pellet, mixed, and then transferred back into PowerBead tube. After addition of solution C1, tubes were incubated at 70°C for 10 min. Then, instead of vortexing for lysis, tubes went through three rounds of bead beating for 2 min (Mini-Beadbeater-96, Biospec Products, Bartlesville, OK) then freezing at -80°C for 2.5 min.

### 16S rRNA Gene Amplicon Sequencing

Extracted DNA was PCR amplified in triplicate. Each 25 μL reaction contained 12.75 μL sterile UltraPure Distilled Water (Invitrogen, Grand Island, NY), 0.5 μL DMSO at a final concentration of 0.28 M, 0.25 μL Herculase II DNA polymerase (Agilent Technologies, Santa Clara, CA), 5 μL buffer, 1 mM dNPTs, 50 ng DNA, and 0.625 μL each of 10 μM bacterial specific primers 926F (5’-AAACTYAAAKGAATTGACGG-3’) and 1392R (5’-ACGGGCGGTGTGTRC-3’) to a final concentration of 0.25 μM with standard 454-Titanium adapters and multiplex identifiers for Lib-L (Roche 454 Sequencing, Madison, WI). PCR reaction conditions included 2 min at 95°C; 30 cycles of 95°C for 20 s, 66.3°C for 20 s, 72°C for 30 s; and a final 3 min elongation at 72°C. PCR products were verified on a 0.8% agarose gel. Pooled triplicates were run on a 2% low-melt agarose gel and cleaned up using a Zymoclean™ Gel DNA Recovery Kit (Zymo Research, Irvine, CA) followed by three rounds of Agencourt AMPure XP beads (Beckman Coulter, Brea, CA) using manufacturer’s protocols. Samples were quantified using a Qubit® Fluorometer (Life Technologies, Grand Island, NY) and pooled to 10^6^ DNA molecules/μL. DNA was sequenced on a GS Junior with FLX Titanium chemistry using previously published long-read modifications [35].

### Amplicon Sequence Processing

Raw data were analyzed using the following steps in mothur v.1.33.3 [36]. Flowgrams were removed that did not contain an exact match to the barcode or primer using the command trim.flows. Then, the command shhh.flows was used to denoise sequences using the flowgrams. Sequences were trimmed to a minimum length of 200 bp using default settings. We aligned unique sequences to the Silva 16S rRNA gene sequence database, version 102, using the default kmer-based search methods [37]. Reads that did not align over the region of interest were removed, and we ran filter.seqs command with “trump =.” to remove excess alignment columns. We removed chimeras identified using UCHIME [38]. Taxonomy was assigned using a mothur-formatted version of the Ribosomal Database Project (RDP) taxonomy training set 9 [39] with a cut-off of 60% identity. OTUs were clustered at 97% identity using the dist.seqs and cluster commands. OTUs were named based on their total abundance across samples. For example, OTU1 was the most abundant OTU overall. Amplicon sequencing data were deposited under Sequence Read Archive accession number SRP059774.

### Statistical Analyses

Comparisons of the qualitative degradation data across dumps and layers were performed in JMP (SAS Institute, Cary, NC) using a survival analysis and the Wilcoxon Group Homogeneity Test. Comparisons of the quantitative degradation data across dumps and layers were performed in JMP using analysis of variance (ANOVA) followed by the Tukey-Kramer honest significant difference (HSD) test.

All sequencing analyses were performed using data rarified to 1701 reads and OTUs classified at 97% similarity. We tested for correlations between the number of reads of an OTU in our sequencing data and the percentage of cellulose degraded by each sample (quantitative data) using ANOVA in JMP. Alpha diversity metrics were calculated in mothur using the summary.single command. We tested for differences in alpha diversity between colonies and dump layers using the Tukey-Kramer HSD test in JMP. Mothur was used to cluster samples with the Morisita-Horn Index using the tree.shared command. A parsimony test to analyze the significance of the cladogram topology with colony, layer, or degradation ability (a categorical ranking based off the level of degradation by the sample) also was performed in mothur. We next analyzed the distance between samples using both the Morisita-Horn Index (dist.shared command) and a weighted Unifrac analysis (unifrac.weighted command) in mothur. The pcoa command in mothur was used to display these distance matrices with a principle coordinate analysis (PCoA). We tested if samples in the PCoAs clustered by colony, layer, or degradation level using an analysis of molecular variance (AMOVA) in mothur. Finally, the corr.axes command was used in mothur to determine the Pearson correlation of the coordinates of the PCoA with the percentage of cellulose degradation and with each OTU. For this correlation analyses, negative values of cellulose degradation relative to the control (within error of zero) were represented as 0%.

### Isolation of dominant community members

Highly cellulolytic communities 1 Top A, 1 Middle E, 1 Bottom E, and 3 Bottom C were dilution plated on the following media types to try to isolate dominant members: M63 cellulose agar: M63 medium (see above) with 15.0 g agar (Amresco, Solon, OH) in 1 L, a 4x4 cm piece of filter paper laid on top of the plate as the sole carbon source; AO Agar: 0.5 g Bacto™ yeast extract (BD, Franklin Lakes, NJ), 0.2 g Bacto™ beef extract (BD, Franklin Lakes, NJ), 0.5 g tryptone (Fisher Scientific, Pittsburg, PA), 2.4 mM sodium acetate (Fisher Scientific, Pittsburg, PA), and 9.0 g agar in 1 L [40]; R2A Agar: 0.5 g Bacto™ yeast extract, 0.5 g proteose peptone #3 (Remel, San Diego, CA), 0.5 g casamino acids (Fisher Scientific, Pittsburg, PA), 0.5 g D-glucose (Research Products International Corp., Mount Prospect, IL), 0.5 g Difco™ soluble starch (BD, Franklin Lakes, NJ), 0.3 g sodium pyruvate (Alfa Aesar, Haverhill, MA), 0.3 g potassium phosphate dibasic, 0.05 g magnesium sulfate, and 15.0 g agar in 1 L [41]; AGS media: 1.0 g arginine monohydrochloride (Sigma-Aldrich, St. Louis, MO), 12.5 g glycerol (Fisher Scientific, Pittsburg, PA), 1.0 g potassium phosphate dibasic, 1.0 g sodium chloride (Fisher Scientific, Pittsburg, PA), 0.5 g magnesium sulfate heptahydrate (Sigma-Aldrich, St. Louis, MO), 0.02 g iron(III) sulfate hexahydrate (Mallinckrodt Chemical Works, St. Louis, MO), 0.001 g copper(II) sulfate pentahydrate (Fisher Scientific, Pittsburg, PA), 0.001 g zinc sulfate heptahydrate (Fisher Scientific, Pittsburg, PA), 0.001 g manganese(II) sulfate monohydrate (Fisher Scientific, Pittsburg, PA), and 15.0 g agar in 1L [42]; Yeast Malt Extract Agar: 4.0 g Bacto™ yeast extract; 10.0 g Bacto™ malt extract (BD, Franklin Lakes, NJ); 4.0 g D-glucose; and 15.0 g agar in 1 L.

Isolated strains were identified through sequencing their 16S rRNA gene using the following procedure with general bacterial primers. A colony of cells was added to 20 μL of microLYSIS®-PLUS (Gel Company, San Francisco, CA), then lysed using the thermal cycler profile: 65°C for 15 min, 96°C for 2 min, 65°C for 4 min, 96°C for 1 min, 65°C for 1 min, and 96°C for 30 s. Two microliters of this lysis solution were mixed with 8.5 μL of water, 1 μL each of 10 μM general bacterial 16S rRNA gene primers 27F (5’-AGAGTTTGATCMTGGCTC-3’) and 1492R (5’-TACGGYTACCTTGTTACGACTT-3’) at a final concentration of 0.4 μM [43], and 12.5 μL of EconoTaq Plus Green 2X Master Mix (Lucigen, Middleton, WI). This mixture was run in a thermal cycler using the parameters: 95°C for 5 min; 30 cycles of 95°C for 1 min, 55°C for 1 min, and 72°C for 2 min; and a final extension of 72°C for 10 min. After verifying amplification using DNA gel electrophoresis with a 0.8% agarose gel, fluorescent dyes were incorporated using reactions consisting of 1 μL of BigDye polymerase (University of Wisconsin-Madison Biotech Center), 1.5 μL of BigDye Buffer, 0.5 μL of primer (either 27F or 1492R) to a final concentration of 0.5 μM, 6.5 μL of water, and 0.5 μL of amplified DNA. Amplification conditions were 95°C for 3 min; 35 cycles of 96°C for 10 s and 58°C for 3 min; and 72°C for 7 min. Samples were sequenced using Sanger sequencing at the University of Wisconsin-Madison Biotech Center (Madison, WI) and analyzed using SeqMan Pro in the DNASTAR Lasergene 11 suite (Madison, WI). The 16S rRNA gene sequence was matched to the RDP database using the options: Type, Isolate, ≥ 1200 bp, good quality [44].

### *Acidovorax* Taxonomy and Growth Assay and Identification

To compare the taxonomy of our *Acidovorax* isolate to the organisms comprising OTU1 in the communities, all 10,803 sequences that clustered into OTU1 in our amplicon data were extracted using the bin.seqs command in mothur and clustered at 100% identity using CD-HIT [45–47]. Clusters containing three or less reads were removed. Representative sequences from each of the seven remaining clusters were aligned in MEGA6 (Muscle, default parameters) [48] with the 16S rRNA gene sequences for all *Acidovorax* type strains and the outgroup *Variovorax paradoxus* from RDP [39]. A maximum likelihood tree was built from the trimmed alignment using RAxML-HPC2 on XSEDE through Cipres with rapid bootstrapping [49,50]. The 16S rRNA gene sequence for this strain, named *Acidovorax* sp. AcolKP-3D, was deposited in GenBank, accession number KT150251.

To test for growth on cellulose, the *Acidovorax* isolate was grown in triplicate in test tubes containing 5 mL of liquid M63 minimal media and supplemented with either 27.8 mM D-glucose, 27.8 mM cellobiose (Sigma-Aldrich, St. Louis, MO), a 1x10 cm strip of filter paper, 0.025 g phosphoric acid swollen cellulose (PASC [51]), or 0.025g crystalline cellulose (Sigmacell, Sigma-Aldrich, St. Louis, MO) as the carbon source. Additionally, the isolate was grown in triplicate in LB broth (Lennox L, Research Products International Corp., Mount Prospect, IL). All cultures were grown at 30°C, shaking at 300 rpm and observed daily for fourteen days. To confirm visual growth observations, 200 μL of each culture was plated onto LB agar plates (LB broth with 15 g agar) after fourteen days. Plates were incubated at 30°C for two days.

## Results

### Electron Microscopy of Leaf-Cutter Ant Refuse Dumps

**Fig 2:**
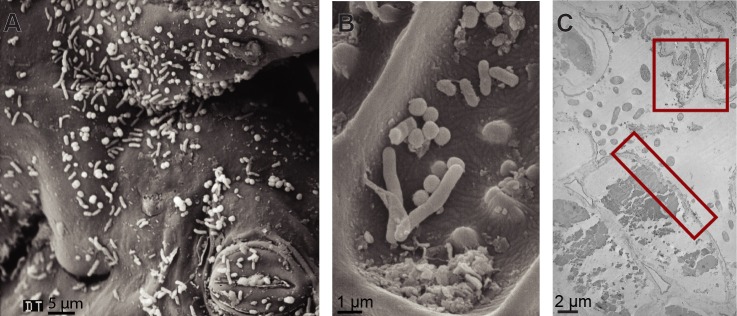
Electron microscopy of leaf-cutter ant refuse dumps. **(A and B)** Scanning electron microscopy shows the ultrastructure of refuse dump leaf material and different bacterial morphologies. **(C)** Transmission electron microscopy shows leaf cells and surrounding bacteria. Red boxes indicate degraded plant cell wall and abnormal, clumped internal cell structure. **Photo credits:** Rolando Moreira Soto.

SEM imaging of leaf material from the top layer of a refuse dump demonstrated that the stoma of the plant and the waxy cuticle remained intact (Fig 2A and 2B, representative images). However, analyses by TEM indicated that the plant cells walls were degraded or partially degraded and the internal structure of the cells was abnormal (red boxes, Fig 2C, representative image). Numerous rod-shaped bacteria and cocci were visible on the plant material and surrounding the plant cells. Interestingly, multiple small clusters of cells were visible, and different morphologies of bacteria appeared to be in close proximity of each other (Fig 2).

### Differences in Cellulolytic Ability across Refuse Dumps

**Fig 3:**
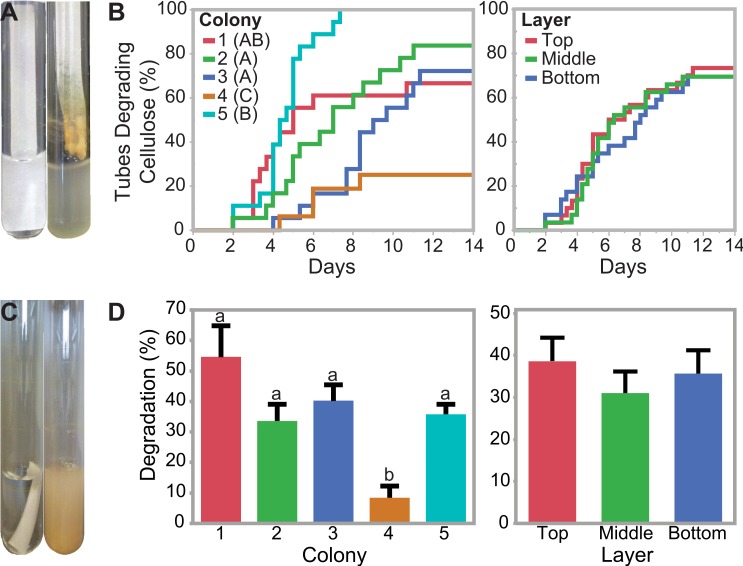
Comparison of degradation ability across colonies and layers. **(A and B**) Qualitative Assay Data. Test tubes containing carbon-free minimal media and a strip of cellulosic filter paper were used to enrich for cellulolytic communities. Failure plots, indicating when the filter paper broke apart in each culture, were fit with Kaplan Meier curves and analyzed using the Wilcoxon method to determine significant differences among colonies (indicated by letters A-C) and layers (no significant differences). (**C and D)** Quantitative Assay Data. Pre-weighed, submerged cellulosic filter paper allowed quantification of cellulose degradation after 10 days. Samples are grouped by colony or dump layer. Error bars represent one standard error from the mean. Significant differences were determined using Tukey’s HSD test and are indicated above the data. **Photo credits:** Gina Lewin (A, C).

Enrichments of refuse dump material on cellulosic filter paper selected for the portion of the refuse dump microbial communities able to grow on and degrade cellulose as the sole carbon source (S1 and S2 Figs; S1 Table). The four fastest microbial communities broke the filter paper in two days (“qualitative assay”; Fig 3A and 3B). In contrast, 26 of the 88 communities tested did not break the filter paper in the 14 day experiment, although 20 of these 26 communities did show visible signs of growth on the filter paper. There were significant differences in the time to degradation when samples were grouped by the ant colony they originated from (Wilcoxon Group Homogeneity Test, p < 0.0001; Fig 3B). All microbial communities from colony 5 degraded the filter paper within 8 days, with an average of 4.6 ± 0.3 days (SE). In contrast, only 4/16 (22%) of the microbial communities from colony 4 degraded the filter paper over the 14 day experiment. Communities from colonies 1, 2, and 3 fell between these two extremes, with 67%, 83%, and 72% degrading the cellulose within 14 days, respectively. In contrast, there were no significant differences in degradation when samples were grouped based on whether they were collected from the top, middle, or bottom layer of the dump (Wilcoxon Group Homogeneity Test, p = 0.8765, Fig 3B).

We quantified the percentage of cellulose degraded in 10 days of growth (“quantitative assay”), and the average cellulose degradation was 35.1 ± 3.1% (Standard Error [SE], Fig 3C and 3D; S1 Table). In the most cellulolytic sample, nearly all cellulose was degraded after 10 days (1 Middle D; 98.6 ±1.8%), and 5 of the 88 samples degraded over 95% of the cellulose. In contrast, 16 of the 88 samples degraded less than five percent of detectable cellulose. Quantitative analyses supported the qualitative assays in demonstrating that the microbial communities from colony 4 were significantly less cellulolytic than communities from the other four refuse dumps (8.4 ± 3.8% degradation [SE]; Tukey-Kramer HSD test, p < 0.01 for all). However, there were no significant differences in the average percentage of cellulose degraded between colony 1, 2, 3, and 5 (54.6 ±10.2%, 33.6 ± 5.5%, 40.3 ± 5.2%, and 35.8 ± 3.3% degradation, respectively; SE). We also did not measure a significant difference between enrichments from the different strata of the dump (ANOVA, F = 0.49, df = 3 and 85, p = 0.6120, Fig 3D).

### Amplicon Sequencing Analysis

To understand how microbial community structure differed among enrichments from different dumps and different degradation abilities, we sequenced the V6-8 region of the bacterial 16S rRNA gene from a subset of enriched samples representing each layer of each dump and a range of degradation abilities (Table 1). We were not able to amplify any fungal sequences from our enrichments using universal fungal primers for internal transcribed spacer sequences (A. Johnson, unpublished data). After sequence processing, there were 104,044 total sequences and 1409 unique sequences across all samples. Sequences per sample ranged from 1701 to 9616 reads (Table 1). To standardize, we subsampled each sample to 1701 reads for all analyses.

**Table 1:** Degradation, sequencing depth, and alpha diversity metrics for sequenced samples at a 97% OTU definition.

Sample^a^	Cellulose Degradation		Degradation Ranking^b^	Number of Reads	Good's Coverage^c^	Observed OTUs^c^	Chao1 Estimator^c^	Inverse Simpson's Metric^c^	Berger Parker Index^c^
	Average	Standard Deviation							
1 Top A	88.3%	1.5%	High	3478	99.1%	55	68	2.8	0.57
1 Top C	95.0%	2.2%	High	4586	99.0%	49	68	3.7	0.38
1 Middle C	-3.9%	1.1%	Low	4045	98.6%	70	102	7.0	0.30
1 Middle E	98.6%	1.8%	High	4183	99.1%	39	64	5.1	0.37
1 Bottom D	4.1%	2.0%	Low	3586	98.9%	54	77	6.8	0.31
1 Bottom E	92.9%	3.6%	High	5380	99.1%	40	61	5.0	0.37
2 Top A	22.8%	8.4%	Medium	4543	99.7%	22	29	3.5	0.47
2 Top B	15.8%	1.8%	Medium	4389	99.6%	27	38	5.7	0.26
2 Middle B	67.1%	5.2%	High	3768	99.3%	50	65	7.4	0.30
2 Middle D	64.2%	4.4%	High	2964	99.4%	35	43	3.2	0.53
2 Bottom A	29.6%	2.6%	Medium	4436	99.0%	50	71	5.5	0.34
2 Bottom D	1.6%	2.1%	Low	2354	98.6%	63	84	6.3	0.24
3 Top A	24.1%	2.6%	Low	1702	98.8%	78	94	14.3	0.18
3 Top F	17.8%	1.7%	Low	4191	98.9%	60	85	12.3	0.16
3 Middle A	41.0%	8.0%	Medium	3485	98.7%	73	93	5.4	0.40
3 Middle E	21.6%	5.0%	Low	4750	98.8%	60	88	10.0	0.24
3 Bottom B	56.7%	0.0%	Medium	5499	98.3%	81	117	12.9	0.21
3 Bottom C	90.6%	5.2%	High	9616	98.7%	63	94	5.8	0.34
3 Bottom D	56.6%	3.6%	Medium	3704	98.7%	66	92	8.7	0.26
3 Bottom F	20.5%	6.3%	Medium	1701	98.9%	60	89	7.2	0.31
4 Top C	49.8%	1.4%	Low	4401	99.1%	54	72	8.4	0.25
4 Middle D	-5.9%	0.6%	Low	4321	98.7%	64	89	2.9	0.57
4 Bottom E	3.1%	2.4%	Low	3253	99.1%	56	79	6.3	0.37
5 Top A	24.6%	1.6%	Medium	3241	98.4%	91	125	14.6	0.17
5 Middle E	36.0%	2.9%	Medium	3709	98.4%	96	129	12.3	0.18
5 Bottom C	52.7%	3.3%	High	2759	99.1%	57	73	4.4	0.45

^a^ Sample name formatting refers to colony sampled, layer of dump, and inoculation replicate.

^b^ Used for analyses that require a categorical variable.

^c^ Metrics calculated using data subsampled to 1701 reads.

**Fig 4:**
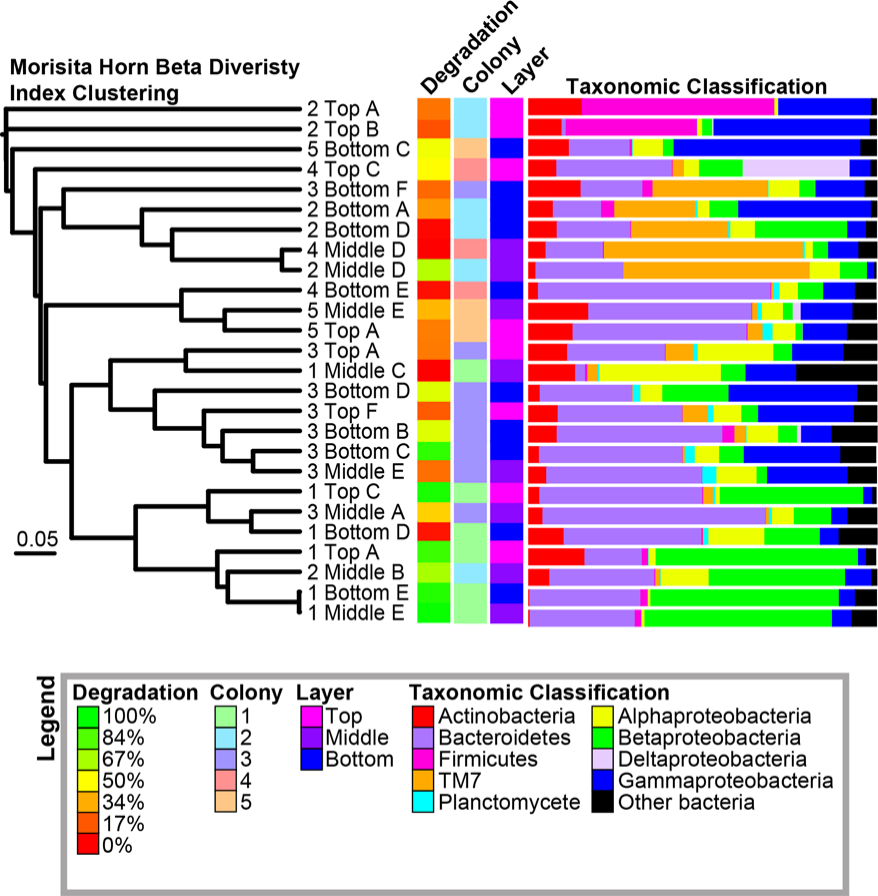
Morisita-Horn Beta Diversity Clustering of Samples. The corresponding percentage of cellulose degradation, colony, layer, and taxonomic classification of OTUs are shown for each sample.

**Fig 5:**
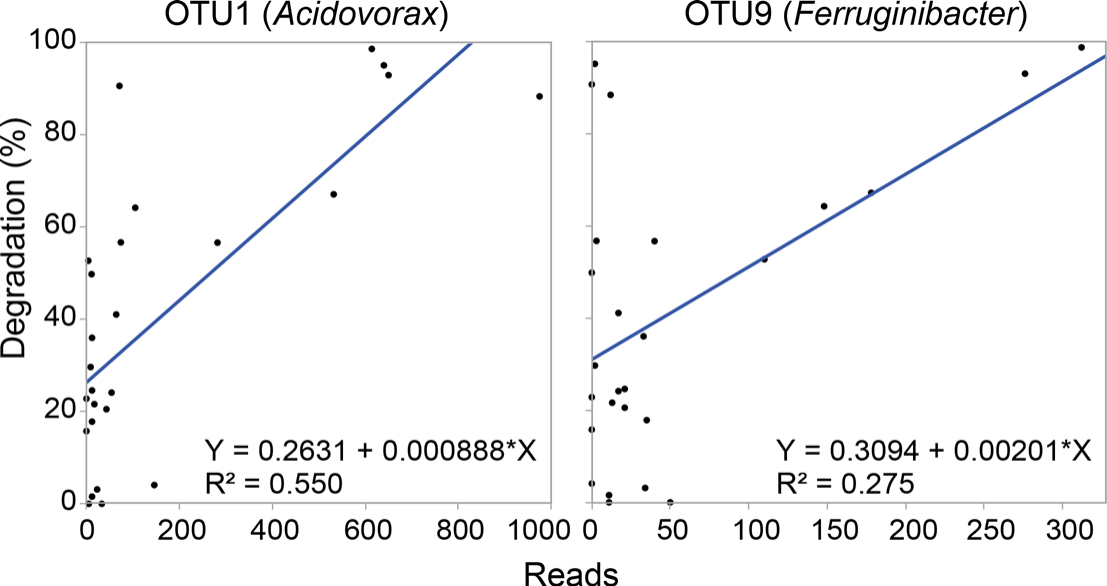
Correlation between number of reads and cellulose degradation by the community for OTU1 (*Acidovorax*) and OTU9 (*Ferruginibacter*) across sequenced samples.

Overall, samples were dominated by Bacteroidetes, Gammaproteobacteria, and Betaproteobacteria (30.7 ± 3.5%, 16.3 ± 2.8%, and 15.7 ± 3.5% of total reads, respectively [SE]; Fig 4; S2 Table). The most abundant operational taxonomic units (OTUs) across all samples were classified as *Acidovorax*, *Leadbetterella*, *Flavobacteriaceae*, *Dokdonella*, and TM7 (Table 2). Twenty of the 30 most abundant OTUs were identified to the genus level, and species from ten of these twenty genera have previously been shown to degrade cellulose (Table 2). We compared the number of reads of the 50 most abundant OTUs to the percentage of cellulose degraded by each sample; the abundances of OTU1 (*Acidovorax*; r^2^ = 0.55; ANOVA, F = 29.38, df = 1 and 25, p < 0.0001) and OTU9 (*Ferruginibacter*; r^2^ = 0.27; ANOVA, F = 9.12, df = 1 and 25, p = 0.0059) were significantly correlated with the percentage of cellulose degraded by the communities (Fig 5).

**Table 2:** Taxonomy of top 30 OTUs across all samples and reports of cellulose degradation^a^.

OTU	Number of Reads	Taxonomy^b^					Report in Literature of Cellulose Degradation	Reference of Degradation
		Phylum	Class	Order	Family	Genus		
OTU1	10803	Proteobacteria	Betaproteobacteria	Burkholderiales	*Comamonadaceae*	*Acidovorax*	Yes	[52,53]
OTU2	8536	Bacteroidetes	Sphingobacteria	Sphingobacteriales	*Cytophagaceae*	*Leadbetterella*	No	
OTU3	7917	Bacteroidetes	Flavobacteria	Flavobacteriales	*Flavobacteriaceae*	unclassified	nd^c^	
OTU4	6971	Proteobacteria	Gammaproteobacteria	Xanthomonadales	*Xanthomonadaceae*	*Dokdonella*	No	
OTU5	6392	TM7	TM7 class incertae sedis	TM7 order incertae sedis	TM7 family incertae sedis	TM7 genus incertae sedis	nd	
OTU6	6200	Actinobacteria	Actinobacteria	Actinomycetales	*Microbacteriaceae* (78)	unclassified	nd	
OTU7	4587	Proteobacteria	Alphaproteobacteria	Caulobacterales	*Caulobacteraceae*	*Asticcacaulis* (77)	Yes	[54]
OTU8	3167	Proteobacteria	Gammaproteobacteria	Xanthomonadales	*Xanthomonadaceae*	*Pseudoxanthomonas* (99)	No	
OTU9	3106	Bacteroidetes	Sphingobacteria	Sphingobacteriales	*Chitinophagaceae* (99)	*Ferruginibacter* (77)	No	
OTU10	3039	Proteobacteria	unclassified	unclassified	unclassified	unclassified	nd	
OTU11	2974	Bacteroidetes	Sphingobacteria	Sphingobacteriales	*Cytophagaceae*	*Sporocytophaga*	Yes	[55,56]
OTU12	2875	Proteobacteria	Betaproteobacteria	Burkholderiales	*Alcaligenaceae*	*Castellaniella*	No	
OTU13	2719	Proteobacteria	Gammaproteobacteria	Xanthomonadales	*Xanthomonadaceae*	*Dyella* (64)	Yes	[57,58]
OTU14	2290	Firmicutes	Bacilli	Bacillales	*Paenibacillaceae* 1	*Cohnella*	Yes	[59]
OTU15	2228	Proteobacteria	Gammaproteobacteria	Xanthomonadales	*Xanthomonadaceae*	unclassified	nd	
OTU16	1707	Bacteroidetes	Sphingobacteria	Sphingobacteriales	*Saprospiraceae*	*Haliscomenobacter*	No	
OTU17	1270	Proteobacteria	Deltaproteobacteria	Myxococcales	*Polyangiaceae*	*Sorangium*	Yes	[60]
OTU18	1083	Proteobacteria	Gammaproteobacteria	Xanthomonadales	*Xanthomonadaceae*	*Stenotrophomonas* (91)	Yes	[61]
OTU19	1027	TM7	TM7 class incertae sedis	TM7 order incertae sedis	TM7 family incertae sedis	TM7 genus incertae sedis	nd	
OTU20	1005	Proteobacteria	Betaproteobacteria	Burkholderiales	*Comamonadaceae*	unclassified	nd	
OTU21	881	Firmicutes	Bacilli	Bacillales	*Paenibacillaceae* 1	*Paenibacillus*	Yes	[62,63]
OTU22	867	Proteobacteria	Betaproteobacteria	Burkholderiales	*Alcaligenaceae*	*Achromobacter* (65)	No	
OTU23	850	Deinococcus-Thermus	Deinococci	Deinococcales	*Trueperaceae*	*Truepera*	No	
OTU24	813	Verrucomicrobia	Opitutae	Opitutales	*Opitutaceae*	*Opitutus*	Yes	[64]
OTU25	792	Proteobacteria	Alphaproteobacteria	Rhizobiales	*Bradyrhizobiaceae*	unclassified (74)	nd	
OTU26	792	Firmicutes	Bacilli	Bacillales	*Paenibacillaceae* 1	*Paenibacillus*	Yes	[62,63]
OTU27	720	unclassified	unclassified	unclassified	unclassified	unclassified	nd	
OTU28	647	Bacteroidetes	Sphingobacteria	Sphingobacteriales	*Sphingobacteriaceae*	unclassified	nd	
OTU29	626	Planctomycetes	Planctomycetacia	Planctomycetales	*Planctomycetaceae*	*Singulisphaera* (91)	No	
OTU30	601	Bacteroidetes	Flavobacteria	Flavobacteriales	*Flavobacteriaceae*	*Ornithobacterium* (69)	No	

^a^ See S2 Table for full taxonomy.

^b^ Number in parentheses indicates confidence score of taxonomic assignment. No number is shown if confidence is 100.

^c^ nd = Not determined because taxonomy not identified to genus level

### Alpha Diversity of Enriched Communities

All samples had high coverage based on Good’s estimator (average = 98.9 ± 0.07% [SE]; range = 98.3% - 99.7%; probability that an additional sequence obtained would already be represented in the dataset). However, the Chao1 richness estimator indicated that 17% to 39% of the OTUs in each sample were not identified by our sequencing (average = 27.5 ± 1.0% [SE]; Table 1). There were slight but significant differences in these diversity indices among some colonies. For example, the samples from colony 2 had significantly lower observed OTUs than those of colonies 3 and 5 (Tukey-Kramer HSD Test, p = 0.0102 and p = 0.0109, respectively), and the samples from colony 2 had significantly lower predicted OTUs (Chao1 richness estimator) than those from colony 5 (Tukey-Kramer HSD Test, p = 0.043). There were no significant differences in diversity metrics among dump layers. Also, there was no correlation between the percentage of cellulose degradation and the observed number of OTUs, the estimated number of OTUs (Chao1 richness estimator), the Inverse Simpson’s Diversity Index, or the Berger Parker Index (dominance measure; Table 1). However, while medium and low degradation samples contained a large range of diversities (Inverse Simpson’s Diversity Index range = 3.5–14.6, average = 8.4 ± 1.3 [SE] and range = 2.9–14.3, average = 8.4 ± 1.3 [SE], respectively), high degradation samples only had low diversity (range = 2.8–7.4, average = 4.7 ± 0.5 [SE]; S3 Fig, Table 1).

### Beta Diversity of Enriched Communities

We clustered samples based on their similarity in community structure using the Morisita-Horn Index, which compares the overlap among samples based on the abundance of each OTU and the total number of OTUs (Fig 4) [65]. Parsimony analysis of the resulting cladogram indicated that the samples clustered significantly based on their source colony (p = 0.005), but not based on their degradation level (high, medium, or low, see Table 1) or layer. Additionally, we mapped the major lineages (phyla or subphyla) found in each sample onto the cladogram (Fig 4). The classification of lineages in each sample matched the clustering patterns of the samples. For example, samples with a high proportion of Betaproteobacteria or TM7 generally clustered together.

In the cladogram, there are samples that cluster but have vastly different cellulolytic abilities (ex. 1 Top C and 1 Bottom D; 4 Middle D and 2 Middle D; 3 Middle E and 3 Bottom D) (Fig 4). To provide insight into relationship between OTU abundance and cellulolytic ability, we performed comparisons on each of these pairs of samples, graphing the abundance of each OTU in the highly cellulolytic sample and the non-cellulolytic sample (S4 Fig). This analysis indicated that in each pair of samples, at least one OTU was identified at high abundance in both communities, likely driving their clustering. However, most of the non-dominant OTUs identified in one sample were absent in the other sample.

**Fig 6:**
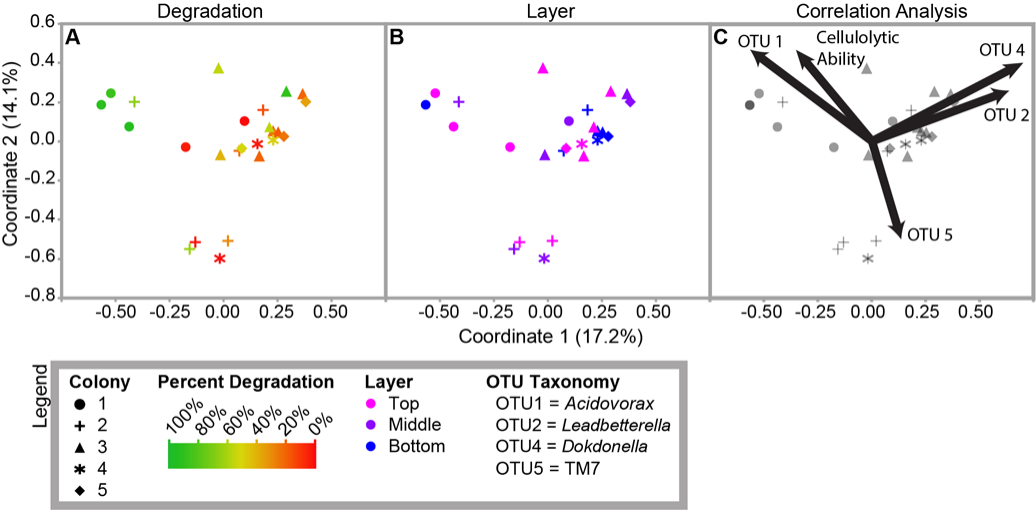
PCoA clustering of Morisita-Horn Diversity Index. Sample shape indicates colony. Sample color indicates degradation (**A**) or layer (**B**). Panel **C** shows the correlation analysis. The vectors indicate the correlation of each OTU and the percentage of cellulose degradation with the principal coordinates shown.

To further analyze the factors that correlated with community diversity, we visualized the Morisita-Horn distance matrix using a PCoA (Fig 6). The resulting matrix significantly clustered by colony (AMOVA, df = 4, F = 2.73, p < 0.001) and degradation level (AMOVA, df = 2, F = 2.39, p < 0.001; Fig 6A). Specifically, highly cellulolytic communities significantly clustered separately from communities with medium or low levels of degradation (AMOVA, df = 1, F = 2.94, p = 0.008 and AMOVA, df = 1, F = 3.39, p = 0.007, respectively). However, there was no significant clustering based on layer (p = 0.44; Fig 6B). We confirmed these patterns by measuring beta diversity using a weighted Unifrac analysis, which quantitatively groups samples by the similarity of their phylogenetic structure (S5 Fig) [66]. Samples clustered significantly when grouped by colony (AMOVA, df = 25, F = 2.4, p < 0.001), as in the Morisita-Horn based clustering. Additionally, when a Bonferroni correction was applied for multiple comparisons, high-degrading samples were significantly different from the low-degrading samples (AMOVA, df = 16, F = 2.33, p = 0.014), but not from medium-degrading samples (df = 16, F = 2.37, p = 0.019).

### Correlation Analysis

We determined the Pearson correlation of the principal coordinates of the PCoA plot with each OTU and with the percentage of cellulose degradation (Fig 6C, S3 Table). The percentage of cellulose degradation correlated positively with coordinate two (p = 0.016). The abundances of OTU1 (*Acidovorax*) and OTU4 (*Dokdonella*) also correlated positively with coordinate two (p = 0.015 and p = 0.04, respectively). In contrast, OTU5’s (TM7) abundances significantly correlated negatively with coordinate two (p = 0.009). Additionally, OTU1’s abundances significantly correlated negatively with coordinate one (p = 0.0028), while OTU4 and OTU2 (*Leadbetterella*) correlated positively with coordinate one (p = 0.000066 and p = 0.00044, respectively). Neither OTU3 (unclassified *Flavobacteriaceae*) or any of the other 30-most abundant OTUs significantly correlated with either coordinate one or two (S3B Table).

### Acidovorax

To understand the role of dominant microbes in the enrichment communities, we isolated 20 strains from highly cellulolytic enrichments on a range of selective and rich media (S4 Table). Of these isolates, the only strain that represented a dominant community member was *Acidovorax* strain AcolKP-3D from the 1 Top A enrichment community, which was isolated on AO agar. Using the Ribosomal Database Project’s seqmatch program, the almost full-length 16S rRNA gene sequence for our strain matched best to *Acidovorax caeni* R-24608, with 90.6% of unique 7-base oligomers shared between the two strains (S_ab score; [67]). Additionally, the 16S rRNA gene sequence of AcolKP-3D was identical to the majority (7391/10803; 68%) of the sequences that comprise the *Acidovorax* OTU1 (S6 Fig).

Because *Acidovorax* sequences were highly detected in our samples and correlated with cellulolytic ability (Table 2, Fig 5, S3 Table), we hypothesized that *Acidovorax* contributes to cellulase production in the community. To test if strain AcolKP-3D could degrade cellulose in isolation, we grew it in the same liquid minimal media as the enrichments with cellobiose, filter paper, PASC, or crystalline cellulose as the sole carbon source, but no growth was observed in any of these cultures after two weeks (S6 Fig). However, the strain showed growth with glucose as the sole carbon source after 4 days and in rich LB liquid media after 1 day (S6 Fig). All growth observations were confirmed using plating onto LB.

## Discussion

Exploring how microbial communities break down plant biomass has important implications for carbon cycling, climate change, and bioenergy research. However, analyses of natural cellulose-degrading communities have proven to be challenging due to their high levels of microbial diversity and complexity. By employing enrichment techniques, we linked community membership and diversity with degradation ability in simplified communities from *A*. *colombica* leaf-cutter ant refuse dumps. Our enrichment strategy allowed us to select for the portion of the community that contributes to cellulose degradation while preserving as many critical inter-species interactions as possible. We observed a significant correlation between the microbial community structure of enriched communities from leaf-cutter ant refuse dumps and their cellulolytic ability. Additionally, community structure correlated significantly with colony of origin.

SEM and TEM images indicated that communities of diverse rod-shaped and cocci bacteria are present on refuse dump material (Fig 2). Additionally, the mixture of intact and degraded plant tissue supports the results of Moreira-Soto et al. [68], who showed that plant cell degradation and the abundance of bacteria increase in the refuse dump relative to the fungus garden. Based on the proximity of the microbes in the images, it is possible that they are interacting, either positively or negatively, as they degrade the plant material.

Our enrichments successfully reduced the diversity of the refuse dump communities while allowing for interactions between organisms. Native refuse dump material has a high level of microbial diversity (average Inverse Simpson’s Diversity Index = 56.6) [25], but in our enriched samples, the average diversity was eight times lower (7.2; Table 1). Community simplification has also been observed in other enrichment-based studies on plant biomass components [10,69]. Additionally, as the communities simplified, certain OTUs began to dominate, as indicated by the increased Berger-Parker Index (average d = 0.16 in native refuse dump material; average d = 0.33 in enriched samples; Table 1) [25]. This decrease in diversity and increase in dominance allowed us to start to understand the ecology of cellulose degradation in communities isolated from refuse dumps.

Our results suggest that there may be a negative correlation between diversity and cellulolytic ability within our samples since high cellulolytic ability was only recorded in low-diversity samples (Table 1, S3 Fig). However, these differences were not significant because low cellulolytic ability was found across a range of high- and low-diversity samples. The correlation between diversity and community function has been a topic of much debate, both in bacteria and eukaryotes [70–72]. In our simplified enrichments, we propose two explanations for the pattern found between diversity and cellulose degradation. Potentially, high cellulolytic ability in low diversity samples was the result of an increased abundance of a few key organisms. This idea is supported by the correlations between the abundances of *Acidovorax* (OTU1) and *Ferruginibacter* (OTU9) with cellulolytic ability (Fig 5). Alternatively, in high diversity samples, negative interactions between organisms could have decreased the level of degradation. Non-cellulolytic organisms may compete with cellulolytic microbes for nutrients including the small oligosaccharides that are released as cellulose is degraded extracellularly. This competition would decrease the growth rates of the cellulolytic microbes and therefore the overall extent of cellulose breakdown.

The taxonomic composition of our enrichments was unique compared with native leaf-cutter ant refuse dumps or fungus gardens and compared with previous enrichment experiments. Different families of Gammaproteobacteria are abundant in the leaf-cutter ant fungus garden (*Enterobacteriaceae*) than in these enrichment communities (*Xanthomondaceae*) [29]. Our communities also show little overlap in abundant OTUs with other plant biomass degradation enrichment experiments [10,17–19], likely because of differences in the carbon sources, growth conditions, and inoculums across these experiments. Similar to native refuse dumps, the majority of our enrichments were dominated by Bacteriodetes, and dominant families including *Comamonadaceae*, *Flavobacteriaceae*, and *Xanthomonadaceae* are abundant in both enriched and native refuse dumps [25]. However, at the genus level, abundant OTUs in these enrichments were detected at very low levels in native refuse dumps, with the exception of *Paenibacillus* [25]. This difference emphasizes that cellulose is not the only carbon source in the refuse dumps, and therefore sequencing of complex natural environments cannot always identify the portion of the community responsible for cellulose degradation.

In this study, there were no significant differences in cellulolytic ability when we grouped samples by layer. In contrast, previous culture-independent analyses of leaf-cutter ant refuse dumps showed that the microbial communities have a predictable pattern of succession between layers [25]. It is possible that the factors that led to the community shift between layers in natural refuse dumps do not vary in our enrichment cultures. For example, in native refuse dumps, there are more aerobes in the upper strata and more anaerobes in lower strata. Since our enrichments were aerobic, we could not detect these differences. Additionally, since cellulose is present in all layers of the dump, cellulolytic microbes may not vary across strata.

We did, however, observe differences in cellulolytic ability and microbial community structure between refuse dumps. While the microbial communities from some dumps could degrade almost all detectable cellulose in 10 days, the communities from other dumps rarely showed signs of cellulose break down (Fig 3). Furthermore, there were differences in microbial community structure among refuse dumps (Figs 4 and 6). Possible factors driving the differences among refuse dumps could include the age of the colony, variation in substrate input, or the temperature, pH, or moisture level in the refuse dump. Also, some refuse dumps may be dominated by cellulolytic fungi instead of bacteria, and since we did not detect any fungi in the enrichments, their contributions would not be represented. Because of the large importance of leaf-cutter ant refuse dumps for carbon turnover, it would be useful to understand if these differences in degradation abilities are steady over time and ecologically relevant.

Interestingly, our results identified multiple communities that were highly similar based on beta diversity metrics but had large differences in cellulolytic ability (Figs 4 and 6, S4 Fig). This observation is counter to the view that similar phenotypic activity correlates with similar community structure. Our analyses indicated that these communities are dominated by a small number of OTUs that are highly abundant in both samples (S4 Fig). Therefore, we predict that the OTU definitions at 97% identity are not always specific enough to differentiate between cellulolytic and non-cellulolytic organisms.

The results from our 16S rRNA gene sequencing suggest that *Acidovorax* spp. may be important for the ability of our enriched communities to degrade cellulose (Table 2, Fig 5), but an *Acidovorax* isolate was not able to grow on cellulose in isolation. Although the strain grew overnight in rich media, it took four days to show growth in glucose media. Furthermore, it did not show signs of growth on cellobiose, filter paper, crystalline cellulose, or the less recalcitrant PASC in the liquid minimal medium used for the enrichments (S6 Fig). These results suggest that *Acidovorax* relies on other member(s) of the community to grow in the enrichments. Possibly, *Acidovorax* is a secondary consumer or scavenger. Alternatively, *Acidovorax* may receive essential nutrients or stimuli for growth and cellulase production from other members of the community.

Many of the other abundant OTUs in our samples were classified in genera that contain known cellulose-degrading strains (Table 2). We hypothesize that these organisms contribute to cellulose degradation in our communities. Although the ability of one species in a genus to degrade cellulose does not mean that other closely-related strains are cellulolytic, cellulases are generally conserved phylogenetically [73]. Therefore, this finding indicates that cellulolytic microbes are enriched for in our experimental samples. Furthermore, the ability to degrade cellulose is a rare trait [1], so it is notable that half of the abundant OTUs were classified in genera that can degrade cellulose. Interestingly, the potentially cellulolytic OTUs were not from well-studied groups of cellulose-degrading microbes. The abundance of poorly studied bacteria in our samples emphasizes the need to explore the diversity of cellulose-degrading microbes that are functionally important in leaf-cutter ant refuse dumps and other natural ecosystems.

A better understanding of the diversity of microbes that contribute to cellulose degradation in the environment is critical for both analyzing the microbial contribution to carbon turnover and for identifying novel enzymes capable of breaking down plant material to produce sustainable cellulosic biofuels. This study shows that microbial communities and their abilities to degrade cellulose can vary significantly, even in seemingly similar environments such as leaf-cutter ant refuse dumps. Therefore, it is necessary to understand the complexities of communities to predict the rate of cellulose degradation and carbon turnover in any specific environment. Cellulose degradation by a small number of model organisms has been extensively studied in laboratory environments, but our understanding of plant biomass decomposition by microbial communities in nature is still limited. This study is an important step in linking the ability to degrade cellulose with the membership and diversity of microbial communities from the leaf-cutter ant refuse dump, and detailed analysis of refuse dumps can serve as a model of community-driven plant biomass degradation in other nutrient-rich environments.

## Supporting Information

S1 Fig Experimental Methods. **(A)** The top (freshest), middle, and bottom (oldest) layers of five refuse dumps were collected. (**B)** Six unique samples from each collection were inoculated into test tubes with cellulose filter paper as the only carbon source in minimal media. These samples were allowed to grow for two weeks and then were transferred into new cultures. (**C)** Transfer cultures included qualitative and quantitative tubes. Qualitative tubes were visually observed daily to determine the number of days necessary for the microbial community to break apart the filter paper. Quantitative tubes were allowed to grow for ten days and then were processed to compare the percentage of cellulose degraded across samples. (**D)** Three days after the filter paper broke apart for each set of qualitative tubes, samples were collected for DNA extraction, 16S rRNA gene amplicon sequencing, and community analysis. A three day period between initial cellulose degradation and DNA extraction ensured that enough growth was present in the tube to analyze the microbial community.
(PDF)

S2 Fig Representative qualitative samples. Images were taken on the day the filter paper broke in half (indicated in parentheses after the sample name) for all cultures except for 3 Top A and the control, which were imaged on day 14.
(PDF)

S3 Fig Relationship between the percentage of cellulose degraded and diversity (Inverse Simpson’s Index) across sequenced samples. Sample shape indicates colony. Sample color indicates layer.
(PDF)

S4 Fig Pairwise comparisons of OTU abundance between samples with similar diversity. Samples were selected with high similarity in diversity but large differences in cellulolytic ability according to the cladogram in Fig 4. The abundance of reads for each OTU is plotted for the indicated samples. We show the best fit line for each plot to indicate the correlation between read abundances of each sample and the y = x line. Abundant OTUs are indicated. See Table 2 or S2 Table for taxonomic classification of each OTU.
(PDF)

S5 Fig PCoA clustering of the weighted Unifrac metric of similarity among samples. Sample shape indicates colony. Sample color indicates degradation (**A**) or layer (**B**).
(PDF)

S6 Fig Phylogeny and growth of an *Acidovorax* isolate. **(A)** Maximum likelihood comparison of the 16S rRNA gene sequence of the AcolKP-3D isolate with all type strains of *Acidovorax*, the outgroup *Variovorax paradoxus*, and amplicon sequencing reads from OTU1. The number of reads matching each sequence of OTU1 is indicated in parentheses. **(B)** Representative images of control and inoculated cultures of AcolKP-3D on LB, glucose, cellobiose, phosphoric acid swollen cellulose (PASC), crystalline cellulose, and filter paper. Images were taken after the number of days indicated. Analysis of growth is indicated, based on visual assessment of liquid cultures and plating.
(PDF)

S1 Table Qualitative and quantitative data for all enrichment communities. (XLSX)

S2 Table Taxonomic classification of reads and OTUs across samples. (XLSX)

S3 Table Correlation analysis of degradation and OTUs with community diversity. (XLSX)

S4 Table Taxonomic identification of isolates. (XLSX)
