# Supplementary material for: Cellulose-Enriched Microbial Communities from Leaf-Cutter Ant (Atta colombica) Refuse Dumps Vary in Taxonomic Composition and Degradation Ability
Source: PLoS One. 2016 Mar 21;11(3):e0151840. doi: 10.1371/journal.pone.0151840 (PMC4801328; doi:10.1371/journal.pone.0151840)
Supplement: S1 Fig — (A) The top (freshest), middle, and bottom (oldest) layers of five refuse dumps were collected. (B) Six unique samples from each collection were inoculated into test tubes with cellulose filter paper as the only carbon source in minimal media. These samples were allowed to grow for two weeks and then were transferred into new cultures. (C) Transfer cultures included qualitative and quantitative tubes. Qualitative tubes were visually observed daily to determine the number of days necessary for the microbial community to break apart the filter paper. Quantitative tubes were allowed to grow for ten days and then were processed to compare the percentage of cellulose degraded across samples. (D) Three days after the filter paper broke apart for each set of qualitative tubes, samples were collected for DNA extraction, 16S rRNA gene amplicon sequencing, and community analysis. A three day period between initial cellulose degradation and DNA extraction ensured that enough growth was present in the tube to analyze the microbial community. (PDF) [file pone.0151840.s001.pdf]

S1 Fig

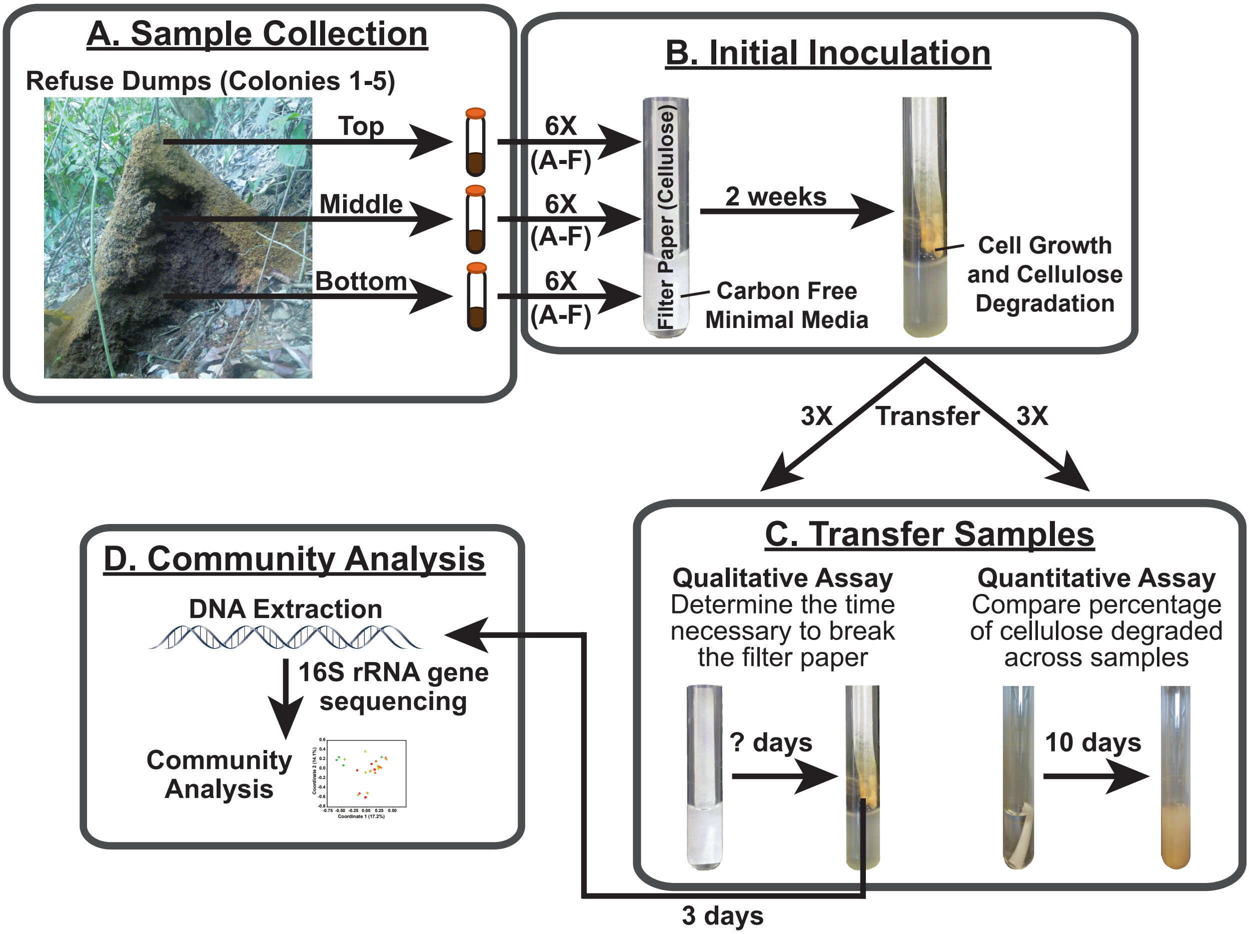

S1 Fig. Experimental Methods. (A) The top (freshest), middle, and bottom (oldest) layers of five refuse dumps were collected. (B) Six unique samples from each collection were inoculated into test tubes with cellulose filter paper as the only carbon source in minimal media. These samples were allowed to grow for two weeks and then were transferred into new cultures. (C) Transfer cultures included qualitative and quantitative tubes. Qualitative tubes were visually observed daily to determine the number of days necessary for the microbial community to break apart the filter paper. Quantitative tubes were allowed to grow for ten days and then were processed to compare the percentage of cellulose degraded across samples. (D) Three days after the filter paper broke apart for each set of qualitative tubes, samples were collected for DNA extraction, 16S rRNA gene amplicon sequencing, and community analysis. A three day period between initial cellulose degradation and DNA extraction ensured that enough growth was present in the tube to analyze the microbial community.
