# Supplementary material for: Cellulose-Enriched Microbial Communities from Leaf-Cutter Ant (Atta colombica) Refuse Dumps Vary in Taxonomic Composition and Degradation Ability
Source: PLoS One. 2016 Mar 21;11(3):e0151840. doi: 10.1371/journal.pone.0151840 (PMC4801328; doi:10.1371/journal.pone.0151840)
Supplement: S2 Fig — Images were taken on the day the filter paper broke in half (indicated in parentheses after the sample name) for all cultures except for 3 Top A and the control, which were imaged on day 14. (PDF) [file pone.0151840.s002.pdf]

S2 Fig

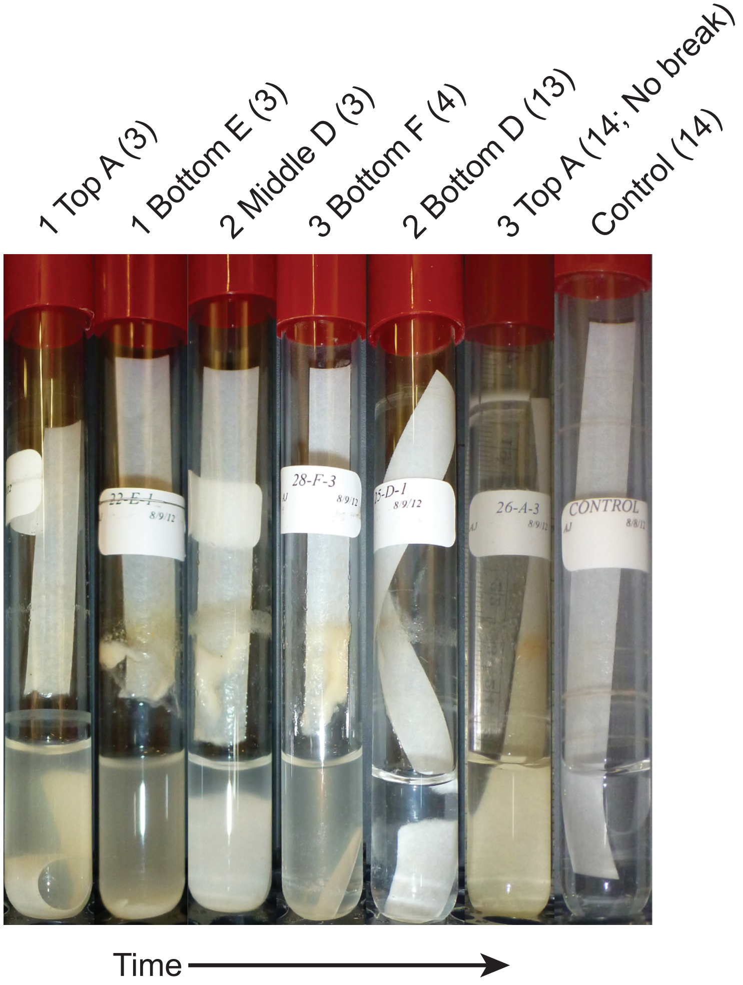

S2 Fig. Representative qualitative samples. Images were taken on the day the filter paper broke in half (indicated in parentheses after the sample name) for all cultures except for 3 Top A and the control, which were imaged on day 14.
