# Supplementary material for: Cellulose-Enriched Microbial Communities from Leaf-Cutter Ant (Atta colombica) Refuse Dumps Vary in Taxonomic Composition and Degradation Ability
Source: PLoS One. 2016 Mar 21;11(3):e0151840. doi: 10.1371/journal.pone.0151840 (PMC4801328; doi:10.1371/journal.pone.0151840)
Supplement: S4 Fig — Samples were selected with high similarity in diversity but large differences in cellulolytic ability according to the cladogram in Fig 4. The abundance of reads for each OTU is plotted for the indicated samples. We show the best fit line for each plot to indicate the correlation between read abundances of each sample and the y = x line. Abundant OTUs are indicated. See Table 2 or S2 Table for taxonomic classification of each OTU. (PDF) [file pone.0151840.s004.pdf]

S4 Fig

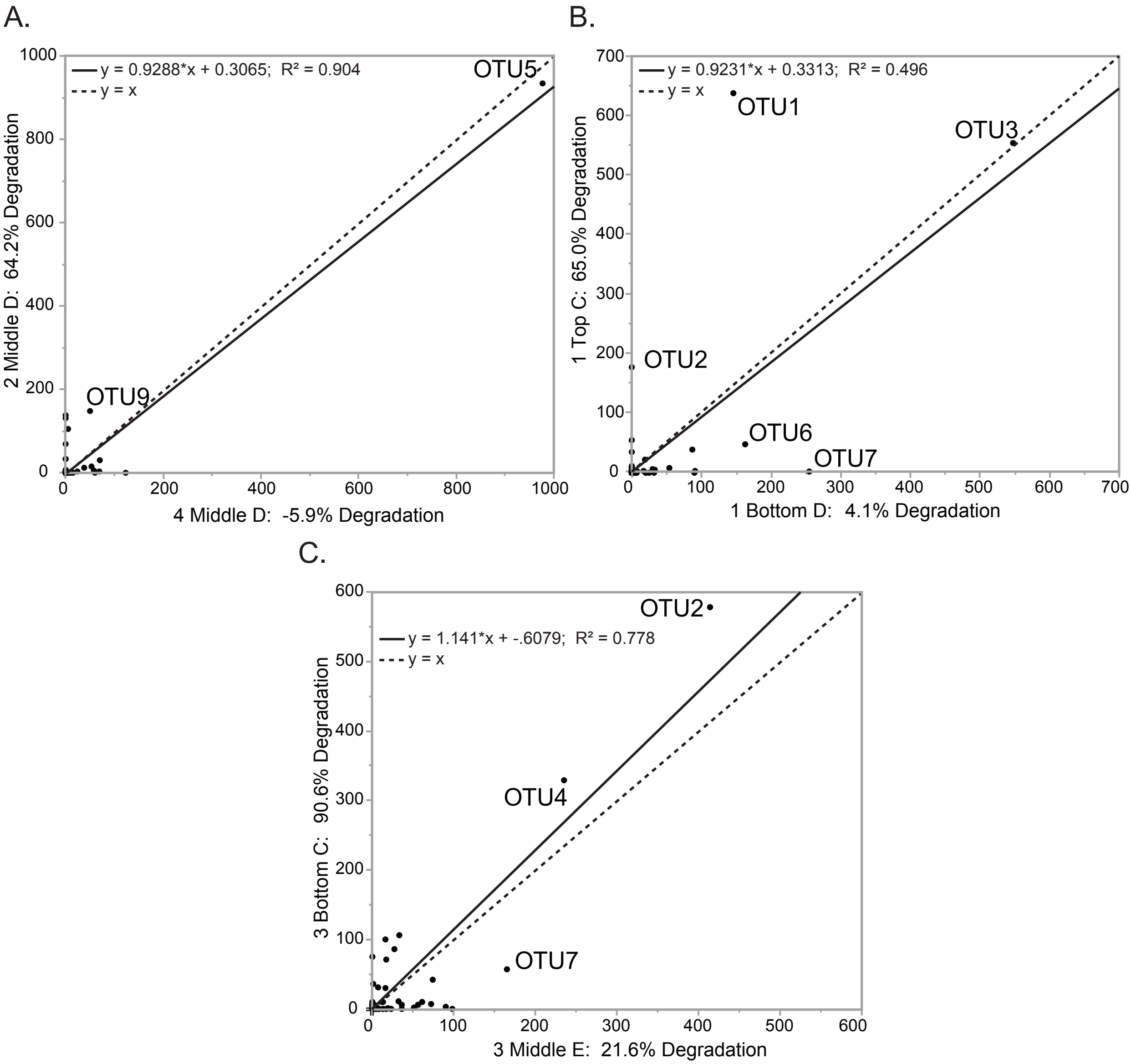

S4 Fig. Pairwise comparisons of OTU abundance between samples with similar diversity. Samples were selected with high similarity in diversity but large differences in cellulolytic ability according to the cladogram in Fig 4. The abundance of reads for each OTU is plotted for the indicated samples. We show the best fit line for each plot to indicate the correlation between read abundances of each sample and the y = x line. Abundant OTUs are indicated. See Table 2 or S2 Table for taxonomic classification of each OTU.
