# Supplementary material for: Cellulose-Enriched Microbial Communities from Leaf-Cutter Ant (Atta colombica) Refuse Dumps Vary in Taxonomic Composition and Degradation Ability
Source: PLoS One. 2016 Mar 21;11(3):e0151840. doi: 10.1371/journal.pone.0151840 (PMC4801328; doi:10.1371/journal.pone.0151840)
Supplement: S5 Fig — Sample shape indicates colony. Sample color indicates degradation (A) or layer (B). (PDF) [file pone.0151840.s005.pdf]

S5 Fig

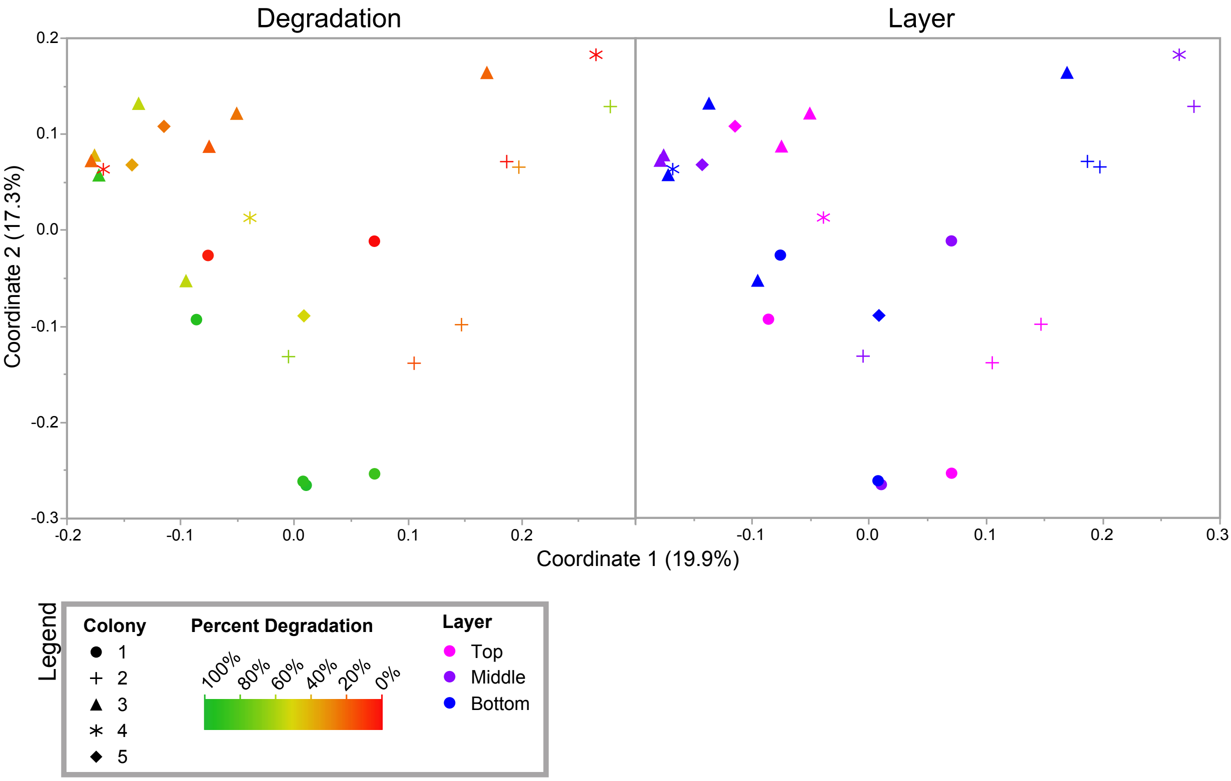

S5 Fig. PCoA clustering of the weighted Unifrac metric of similarity among samples. Sample shape indicates colony. Sample color indicates degradation (A) or layer (B).
