# Supplementary material for: Cellulose-Enriched Microbial Communities from Leaf-Cutter Ant (Atta colombica) Refuse Dumps Vary in Taxonomic Composition and Degradation Ability
Source: PLoS One. 2016 Mar 21;11(3):e0151840. doi: 10.1371/journal.pone.0151840 (PMC4801328; doi:10.1371/journal.pone.0151840)
Supplement: S6 Fig — (A) Maximum likelihood comparison of the 16S rRNA gene sequence of the AcolKP-3D isolate with all type strains of Acidovorax, the outgroup Variovorax paradoxus, and amplicon sequencing reads from OTU1. The number of reads matching each sequence of OTU1 is indicated in parentheses. (B) Representative images of control and inoculated cultures of AcolKP-3D on LB, glucose, cellobiose, phosphoric acid swollen cellulose (PASC), crystalline cellulose, and filter paper. Images were taken after the number of days indicated. Analysis of growth is indicated, based on visual assessment of liquid cultures and plating. (PDF) [file pone.0151840.s006.pdf]

S6 Fig

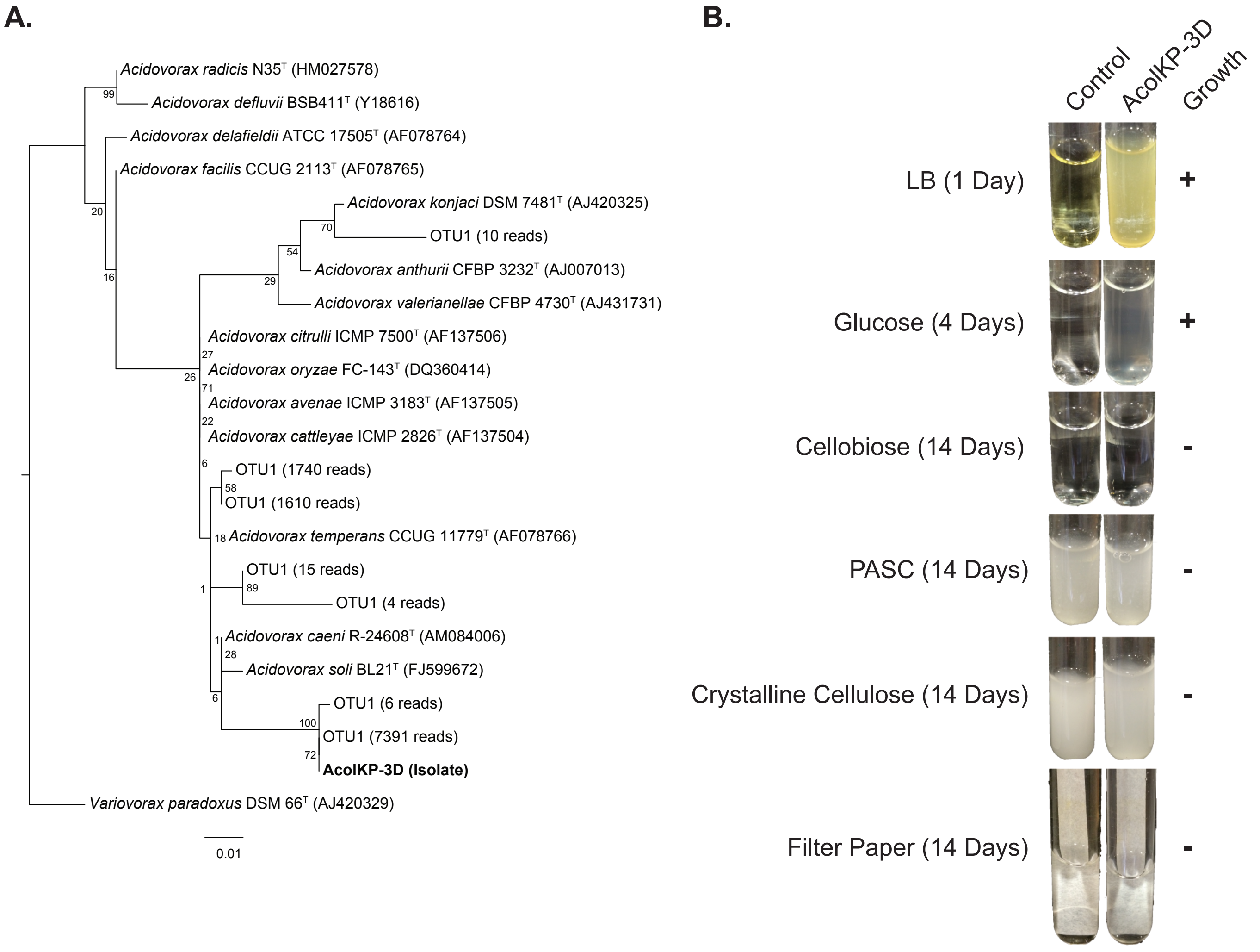

S6 Fig. Phylogeny and growth of an *Acidovorax* isolate. (A) Maximum likelihood comparison of the 16S rRNA gene sequence of the AcoIKP-3D isolate with all type strains of *Acidovorax*, the outgroup *Variovorax paradoxus*, and amplicon sequencing reads from OTU1. The number of reads matching each sequence of OTU1 is indicated in parentheses. (B) Representative images of control and inoculated cultures of AcoIKP-3D on LB, glucose, cellobiose, phosphoric acid swollen cellulose (PASC), crystalline cellulose, and filter paper. Images were taken after the number of days indicated. Analysis of growth is indicated, based off visual assessment of liquid cultures and plating.
